# Classification of Articulator Movements and Movement Direction from Sensorimotor Cortex Activity

**DOI:** 10.1038/s41598-019-50834-5

**Published:** 2019-10-02

**Authors:** E. Salari, Z. V. Freudenburg, M. P. Branco, E. J. Aarnoutse, M. J. Vansteensel, N. F. Ramsey

**Affiliations:** UMC Utrecht Brain Center, Department of Neurology and Neurosurgery, University Medical Center Utrecht, Utrecht, The Netherlands

**Keywords:** Brain-machine interface, Neuromuscular disease

## Abstract

For people suffering from severe paralysis, communication can be difficult or nearly impossible. Technology systems called brain-computer interfaces (BCIs) are being developed to assist these people with communication by using their brain activity to control a computer without any muscle activity. To benefit the development of BCIs that employ neural activity related to speech, we investigated if neural activity patterns related to different articulator movements can be distinguished from each other. We recorded with electrocorticography (ECoG), the neural activity related to different articulator movements in 4 epilepsy patients and classified which articulator participants moved based on the sensorimotor cortex activity patterns. The same was done for different movement directions of a single articulator, the tongue. In both experiments highly accurate classification was obtained, on average 92% for different articulators and 85% for different tongue directions. Furthermore, the data show that only a small part of the sensorimotor cortex is needed for classification (ca. 1 cm^2^). We show that recordings from small parts of the sensorimotor cortex contain information about different articulator movements which might be used for BCI control. Our results are of interest for BCI systems that aim to decode neural activity related to (actual or attempted) movements from a contained cortical area.

## Introduction

Even though speech has been suggested to involve more than one hundred muscles^1–3^ and the control of articulator movements is very precise and complex, after several years of training during childhood most people can speak without much effort. However, for some people who have very severe forms of paralysis the control of the articulators is completely absent and communication by speech is severely impaired and sometimes even impossible^4–6^. This situation, where people have intact cognition but no or very limited ways to communicate due to paralysis, is called Locked-In Syndrome (LIS)^4^. People with LIS may benefit from current developments in assistive communication technology. A brain-computer-interface (BCI) system for instance, is a system that enables people with LIS to control a computer without muscle involvement, using only brain activity. Brain activity from the sensorimotor cortex (SMC) has been the focus of a large part of BCI research as it can be used as a source of control signals and it has been shown that neural activity related to hand movements can be reliably recorded with implanted electrodes^7–9^, and used for BCI control in a home-use setting^10^.

Recent studies have however, also investigated the neural basis of speech movements for BCI control. Conceptually, the direct identification of internally spoken words using the SMC activity generated by the associated attempted articulator movement would provide people with LIS with a highly intuitive and muscle-independent manner of communication. Several studies have now demonstrated that it is indeed possible to decode phonemes, sounds and words using SMC activity measured with electrocorticographic (ECoG) electrodes (e.g.^11–16^). In addition, whole sentences could be reconstructed based on neural activity during natural speech (word accuracy around 75% in a set of 10 words^14^). Yet, solving the problem of reliable and real-time speech decoding, is highly complex due to the high dimensionality of speech and may therefore benefit from a thorough understanding of the representation of the involved articulators in the SMC and of an assessment of the subareas of the SMC that provide the best classification results. Recent work showed a high level of speech decoding from perisylvian regions combined, including auditory cortex responding to the generated speech^17^. Given lack of auditory feedback in LIS, an earlier functional magnetic resonance imaging (fMRI) study by Bleichner *et al*.^18^ investigated the sensorimotor cortex neural activity patterns selectively for isolated movements of different articulators (larynx, tongue, lips and jaw) and showed that these can be distinguished (i.e. classified) from each other with high accuracy (ca. 90%). However, in contrast to fMRI, which measures throughout the whole depth of the SMC (where electrodes for BCI implants cannot reach), ECoG measures from the surface of the cortex and it remains to be determined therefore if similar accuracy levels can be achieved for articulator movement classification using ECoG surface electrodes. In addition, it has been suggested that during speech different positions of the *same* articulator result in different SMC activity patterns^19,20^, although more research is needed to investigate the extent of this.

Therefore, we investigated if movements of different articulators (lips, tongue, jaw, larynx), but also different movements of the same articulator can be distinguished from one another based on sensorimotor cortex activity with ECoG recordings. For the latter we chose the tongue since this is an important articulator in differentiating sounds^21,22^ and it can be predicted which sound somebody said based solely on information about tongue position^23^. Furthermore, the tongue can easily be moved in different directions. In addition, we investigated which SMC areas contained the most information about different articulator and tongue movements. Finally, as it is important to minimize electrode-grid sizes to keep surgical risks low, we investigated the minimal grid size at which accurate classification was possible.

We analysed high density ECoG recordings from the sensorimotor cortex of 4 participants with epilepsy. A template matching classification method with 10-fold cross-validation was used to classify different articulator movements and different tongue movement directions based on spatial or spatio-temporal SMC activity patterns. We focused on high-frequency band (HFB; 60–130 Hz) power changes in the SMC, since HFB power has been shown to correlate well with articulator movements^24,25^ and has been linked to neural firing^26–28^.

## Methods

### Participants

Four patients (A–D, age 19–41 y; median 34.5 y, 2 females) participated in this study, while they were treated in the University Medical Center Utrecht for epilepsy. Subdural clinical-ECoG electrodes (interelectrode distance 1 cm) were implanted sub-chronically (subject A, B and D) or were used during awake surgery (subject C) to record neural signals for clinical purposes. For research purposes, a non-clinical high-density (HD) electrode grid was placed (sub-chronically or temporarily during awake surgery) over a clinically non-relevant area (the sensorimotor cortex) with the consent of the participants. HD electrodes were 1 or 1.17 mm in diameter with a 3- or 4-mm inter-electrode distance (Table 1). For the current study, only signals recorded with the HD electrodes were used in the analyses.

**Table 1:** Recording details.

Subject	A	B	C	D
**Tasks performed**				
Articulator Task run 1	X	X	X	X
Articulators Task run 2	X	X		
Tongue Task (inside mouth) run 1	X	X		X
Tongue Task (inside mouth) run 2		X		
Tongue Task (outside mouth)	X	X		
**Electrode diameter (mm)**	1	1.17	1	1
**Inter-electrode distance (mm)**	4	4	3	3
**Number of recorded electrodes**	64	128	128	128

In the top section, we show which subject did which task. In the bottom section, we indicate per subject the details of the electrode grids.

This study was conducted in accordance with the Declaration of Helsinki (2013) and was approved by the ethics committee of the University Medical Center Utrecht. Written informed consent was given by all subjects.

### Tasks

The participants performed two tasks. First, we wanted to see if we could replicate earlier fMRI findings by Bleichner *et al*.^18^ showing that movements of different articulators can be classified from SMC neural activity. Therefore, in the first task (the ‘Articulator Task’), subjects were asked to make four different articulator movements. The movements were (1) pursing the lips, (2) clenching the teeth, (3) moving the tongue from left to right behind the teeth, (4) making a ‘mmm’ sound. These movements involve the lips, jaw, tongue and larynx, respectively, and were chosen to be similar to those used by Bleichner and colleagues^18^. For the patients with subchronic electrode implants, the task was presented on a computer screen at a comfortable distance from the participants. During the intraoperative recordings, the task was presented on a tablet that was attached to a pole such that it was clearly visible to the participant. A trial started with a 1500 ms visual cue, being the Dutch word ‘lippen’ (lips), ‘tanden’ (teeth), ‘tong’ (tongue), or ‘mmm’. This visual cue instructed the subjects to start the movement and hold it for as long as the cue was visible. Movement trials were interleaved with rest trials, indicated by a ‘-’ symbol, in which the subject was instructed not to move and keep the articulators in a comfortable, neutral position. Trials were followed by an inter-trial interval of 1500 ms (subjects A, B & D) or 2000 ms (subject C) during which a fixation cross was presented. Each movement was repeated 20 times in random order as were the rest trials (20 in total).

In the second task (the ‘Tongue Task’), the subjects were instructed to make four different tongue movements inside the mouth. The movements were (1) up, (2) down, (3) left and (4) right from rest position. This task was similar to the first task except that the visual cues now were arrows pointing in each direction. During the rest trials, subjects were instructed not to move the tongue in any specific direction but keep it in a comfortable position at the bottom of the mouth cavity.

Some subjects performed some additional runs of the experiments, see Table 1. Subject C only performed one task (the Articulator Task) due to limited time in the intraoperative setting. Subjects A & B performed the Tongue Task also once with the tongue outside the mouth during which they continuously stuck out their tongue and moved it in the indicated direction.

### Data acquisition & preprocessing

The sampling frequency of the neural signal recordings was 512 Hz (subject A; Micromed, Treviso, Italy) or 2000 Hz (subjects B, C & D; Blackrock Microsystems LLC, Salt Lake City, USA). In total, we recorded from 64 electrodes for subject A and from 128 electrodes for subjects B-D. For all subjects except subject C, the electrode positions were identified from a post-operative computed tomography (CT) scan and were subsequently plotted over a 3D surface of the subjects pre-operative magnetic resonance imaging (MRI) scan^29,30^. For subject C, there was no CT scan as the data was recorded intraoperatively and electrode positions were determined using the correlation between the HFB power pattern over the grid (during rest) and the underlying anatomical structure (e.g. the location of sulci or blood vessels) to estimate the electrode positions. This method has been validated for localization of HD electrodes^31^.

We used Matlab software (The Mathworks, Inc., Natick, MA, USA) for all data analysis. First, flat or noisy electrodes were removed from further analysis similar as in Salari *et al*.^32^. For the remaining electrodes, we applied a notch filter for the removal of line noise and harmonics thereof. For removal of any remaining artefacts, a common-average re-referencing was applied. Subsequently, we used a Gabor wavelet^33^ to calculate the high frequency band (60–130 Hz) power per sample point for each included electrode. The Gabor wavelet was calculated with a full width half maximum (fwhm) of 4 wavelets per frequency in bins of 1 Hz for all frequencies between 60 and 130 Hz. The Gabor wavelet results were subsequently log transformed (10*log10) and averaged over frequencies to create the HFB power signal per electrode. Finally, we determined for each electrode if it was responsive to the task. To that purpose, we computed, per included electrode, the r^2^-value by correlating the mean activity levels during active periods (from cue until the end of a trial) and rest periods (rest trials) with the task design. This was done for each movement separately. We determined the significance level of the r^2^-value of each electrode by using a Monte Carlo distribution (shuffling active and rest labels using 10.000 permutations, Alpha = 0.05, false discovery rate corrected). This was done once with all trials to determine the spatial activity pattern for each movement and again for electrode selection during classification but only on the training set due to the 10-fold cross-validation procedure (see below for details). Electrodes that did not show a significant HFB power response to any of the movements of a task were removed from analysis of that task. For classification, we used a 10-fold cross-validation, meaning that for each cross-validation iteration we selected the significant electrodes only based on the training set (not including the test trials). By doing so, the electrodes that were non-responsive to the task were excluded only on the basis of the training set. This was done to make sure that feature selection and model training was not affected by the test set (the trials that had to be classified).

### Classification procedures

For classification, we used a template matching procedure (see below for details) that is similar to what has been used successfully before for the classification of speech sounds and hand gestures^7,15^. First, we smoothed the signal of each electrode with a moving average window of 0.5 seconds around each sample point, which has been shown previously to be an optimal setting for classification of movements from ECoG neural recordings^34^. Note that analyses with shorter smoothing kernels (down to 0.1 seconds) did not improve classification results and are therefore not presented here. Next, we z-scored the smoothed signal and epoched the result in trials of 2 seconds, each starting at cue onset. We then performed two types of template matching classification procedures with a 10-fold cross-validation as explained below.

First, we discarded time and created a spatial power template for each of the movements by averaging the power signal of each electrode over the whole trial period (i.e. spatial classification). Subsequently, for the 10-fold cross-validation, trials were split into 10 equal sets of trials (each set containing 8 trials, 2 of each class) and each set was taken out of the total data set once and used for classification, while the remaining trials were used for creating the spatial templates of the four conditions by averaging (per electrode) the values of all remaining trials within one class. These templates were then correlated with the trials that were taken out and the template with the highest correlation to each trial was chosen in a winner-takes-all fashion. This was repeated until all sets were classified. Finally, the classification accuracy for each set was calculated by counting the number of correctly classified trials divided by the total number of trials. We then calculated the mean accuracy and standard deviation over cross-validation iterations.

Second, to investigate whether including temporal information would lead to better classification scores, we repeated the classification procedure method but without averaging the power over the trial period, thereby including temporal information in the classification (i.e. spatio-temporal classification).

### Anatomical localization of informative electrodes

To investigate where exactly the most informative electrodes for articulator and tongue movement classification were located, we used a random search procedure in which we randomly chose a subset of electrodes to classify from and repeated this 5000 times. Subsequently, for each electrode we calculated what the average accuracy was if that electrode was part of the electrode set. These scores were subsequently z-scored to find the hotspots of electrodes that were the most informative. We investigated how the number of iterations for this procedure influenced the final pattern of most informative electrodes by determining the informative-electrode-pattern at each iteration and subsequently calculating the mean correlation of that pattern with the pattern of all subsequent iterations. This shows how much the pattern changes with each extra iteration. That is, with only a few iterations the result will be highly variable since only a few of the possible electrode combinations have been tested. However, with more iterations, the result will differ less with the result of the previous iteration as more electrode combinations have been tested. The less the pattern changes, the more reliable the pattern result is. We found that 5000 iterations gave a reliable result, see Supplementary Fig. S1.

### Anatomical scale of informative areas

For an eventual BCI implantation, it is desirable to keep the size of the implanted grid as small as possible, which reduces surgical risk. Therefore, we wanted to assess not only where the most informative electrodes where located given the current grid sizes but also what the classification accuracy would be for smaller grids. Therefore, we used a searchlight approach in which we started with a small grid size (only 1 electrode) and then increased the grid size step-by-step, each time with one extra row and column of electrodes (successively the grid size was 1 × 1, 2 × 2, 3 × 3, 4 × 4 etc.), until the real grid size was reached. This was done to find the minimal size of the area that was needed for accurate classification, given that electrodes could not be anywhere, but were restricted to be closely located to each other as would be the case with smaller ECoG grids. Per grid size and per location, we calculated the accuracy score (only for spatial classification). We determined the maximum accuracy score per grid size (best of all sampled locations) to assess the minimally required grid size for accurate classification. Note that for both the searchlight and random search analyses, we used algorithms that were based on leave-one-trial-out cross-validation as these algorithms were designed to be able to work with little data.

### Topographical organization of the articulators

While previous research has indicated overlap between the areas that are active during movements of different articulators^35^, the articulators are also known to show a topographical organization in the SMC^25,35^. We used a procedure described by Bruurmijn and colleagues^36,37^ to normalize the SMC of each subject into an isotropic coordinate system to further investigate the topographical organization of the representation of the different articulator movements. We created a group average activity pattern of the recorded SMC for each articulator movement and each tongue movement. This was done by plotting for each electrode, the r^2^ value for each movement within the normalized coordinate system. In addition, we made a winner-takes-all plot, indicating for each electrode, which movement had the highest r^2^-value. Only electrodes with a r^2^-value of more than 0.2 in at least one of the conditions were used for this analysis.

### Ethical approval

All procedures performed in studies involving human participants were in accordance with the ethical standards of the institutional and/or national research committee and with the 1964 Helsinki declaration and its later amendments or comparable ethical standards.

### Informed consent

Informed consent was obtained from all individual participants included in the study.

## Results

In general, subjects did not report to have any difficulty performing the tasks, except for subject A who reported that the Articulator Task was a little fast. We did not exclude any trials based on performance, however, since performance of most of the movements had to be done with the mouth closed, and performance could not be accurately assessed.

During the first run, 60.94% (39/64), 80.47% (103/128), 87.50% (112/128), and 84.38% (108/128), of all electrodes of subjects A-D, respectively, showed a significant correlation with at least one of the conditions of the Articulator Task. Subject A & B performed this task twice and for the second run 71.88% (46/64) and 92.97% (119/128) of the electrodes were significant for subjects A & B, respectively. For the Tongue Task, 53.12% (34/64), 68.75% (88/128), and 64.84% (83/128) of the electrodes of subjects A, B and D showed a significant correlation with the task. Note that subject C did not perform this task. Subject A & B also performed a run with the tongue outside the mouth, resulting in 51.56% (33/64) and 68.75% (88/128) of the electrodes showing a significant correlation with the task. Subject B performed a second run with the tongue inside the mouth and 64.06% (82/128) of the electrodes were significant.

### Spatial classification accuracy

**Figure 1:**
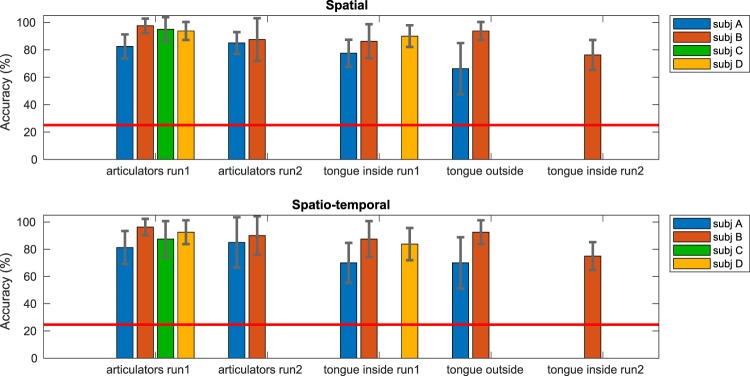
Classification accuracies. The mean classification accuracies (over cross-validation iterations) and standard deviations are shown for each subject (**A–D**, in different colors), per run. Classification was based on all electrodes that showed a significant relation to the task. Runs are indicated on the x-axis and the classification accuracy is indicated on the y-axis. In the upper panel, the spatial classification accuracies are shown. In the lower panel, the accuracies for spatio-temporal classification are given. The red line indicates the chance level of 25%. Note that not all subjects performed two runs and subject (**C**) only performed the articulator movement task.

When classification was based on only spatial features, average classification accuracy (mean over the four subjects) for the Articulator Task was 92.19% (SD = 6.64, n = 4) for run 1 and 86.25% (SD = 1.77, n = 2) for run 2 (Fig. 1). For the Tongue Task the classification accuracy was 84.58% (SD = 6.41, n = 3) for run 1. Only one subject did a second run of this task and had a classification accuracy of 76.25%. For the Tongue Task with the tongue outside the mouth, the mean accuracy was 80.00% (SD = 19.45, n = 2). Classification was significant (p < 0.001) for all subjects, runs and tasks. See Supplementary Fig. S2 for the confusion matrices.

### Spatio-temporal classification accuracy

When classification was based on spatio-temporal features, mean classification accuracy was 89.38% (SD = 6.50, n = 4) for run 1 and 87.50% (SD = 3.54, n = 2) for run 2 (Fig. 1) for the Articulator Task. For the Tongue Task the classification accuracy was 80.42% (SD = 9.21, n = 3) for run 1. The one subject who did a second run had a classification accuracy of 75.00%. For the Tongue Task with the tongue outside the mouth the mean accuracy was 81.25% (SD = 15.91, n = 2). Classification was significant (p < 0.001) for all subjects, runs and tasks. See Supplementary Fig. S3 for the confusion matrices.

### Anatomical localization

Since most participants performed only one run of each task, the next results are based on the first run of each participant. For the participants who performed multiple runs, however, the results of the second run are similar to that of the first, as displayed in the Supplementary Material (Figs S4 & S5).

**Figure 2:**
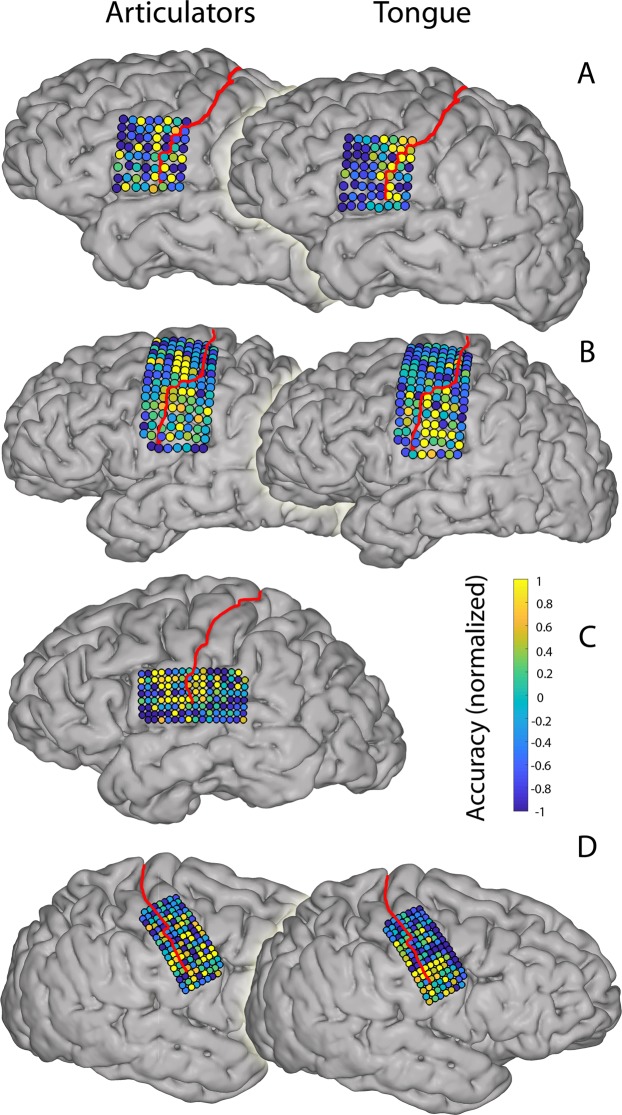
Most informative electrodes. The most informative electrodes are shown for each subject (**A–D**) and for each task. Informative electrodes were determined by a random search procedure. Colors indicate the normalized average accuracy score of this procedure. The warmer the color the higher the classification was on average if that electrode was included. Results are based on the first run of each task, per participant. See Supplementary Material for the data of the other runs.

The most informative electrodes for classification of articulator movements were more spread out over the sensorimotor cortex than the electrodes that were most informative for classification of tongue movements, which were clustered in the central aspects of the vSMC (Fig. 2).

**Figure 3:**
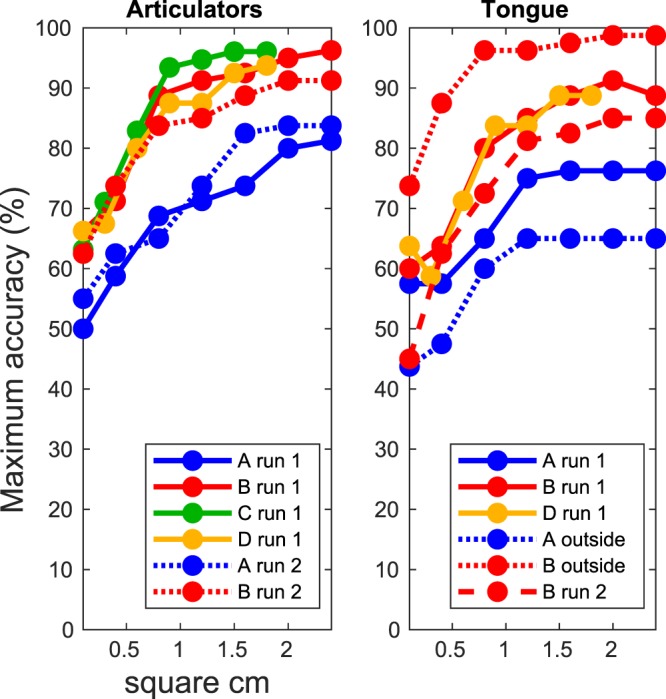
Size of cortical area required for accurate classification. The maximum classification accuracy (y-axis) is shown for different sizes of cortical surface area (x-axis), for each subject. This was calculated by using a search light approach in which we increased the sampled area of the search light and for each size computed the highest accuracy.

Results of the searchlight procedure show for each cortical area sampling size the maximum classification accuracy score that can be obtained. For the Articulator Task, the cortical area necessary for a subject to reach more than 70% accuracy was on average 0.62 cm^2^ (SD = 0.40, n = 4), and to reach more that 80% on average 1.18 cm^2^ (SD = 0.83, n = 4). For the Tongue Task, 0.87 cm^2^ (SD = 0.31, n = 3) was needed to reach 70% accuracy and 1.05 cm^2^ (SD = 0.21, n = 2) to reach 80% accuracy (Fig. 3; note that subject A did not reach 80% for tongue movement directions). These results suggest that the cortical foci (i.e. electrodes) that contain information about movements of different articulators or different tongue directions are relatively close together.

**Figure 4:**
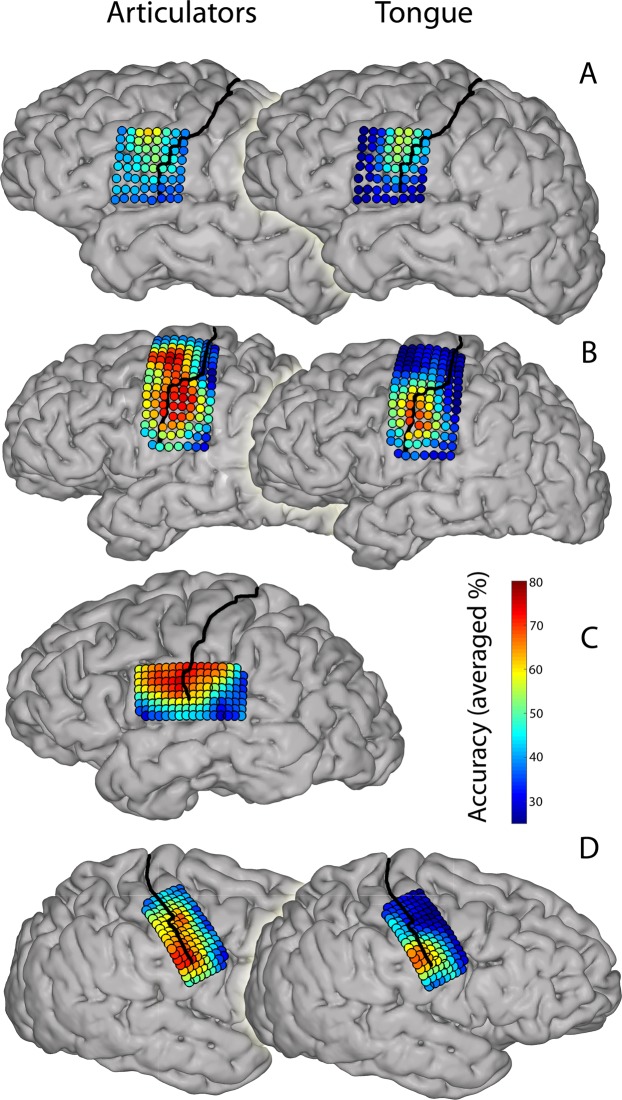
Search light results. The most informative areas are shown for each subject (**A–D**) and for each task. Informative areas were determined by a search light procedure (a 3 × 3 grid size was used for the current figure). For this plot, we chose a search light of three electrode rows and columns, corresponding to approximately 12 × 12 mm for subject (**A,B**) and 9 × 9 mm for subjects (**C,D**). Colors indicate the average accuracy score of this procedure. The warmer the color the higher the classification was on average. Results are based on the first run of each task and participant. See Supplementary Material for the data of the other runs.

Visual inspection of the location of the most informative electrodes, as obtained in the searchlight procedure, shows that the cortical area that *can* be used for classification of different articulator movements is larger than the area for different tongue direction movements (Fig. 4). Some areas contribute both to articulator and tongue movement classification, which suggests that within the same small ECoG grid, classification of both different articulator movements and different tongue movement directions can be accurate.

### Topographical organization of the articulators

**Figure 5:**
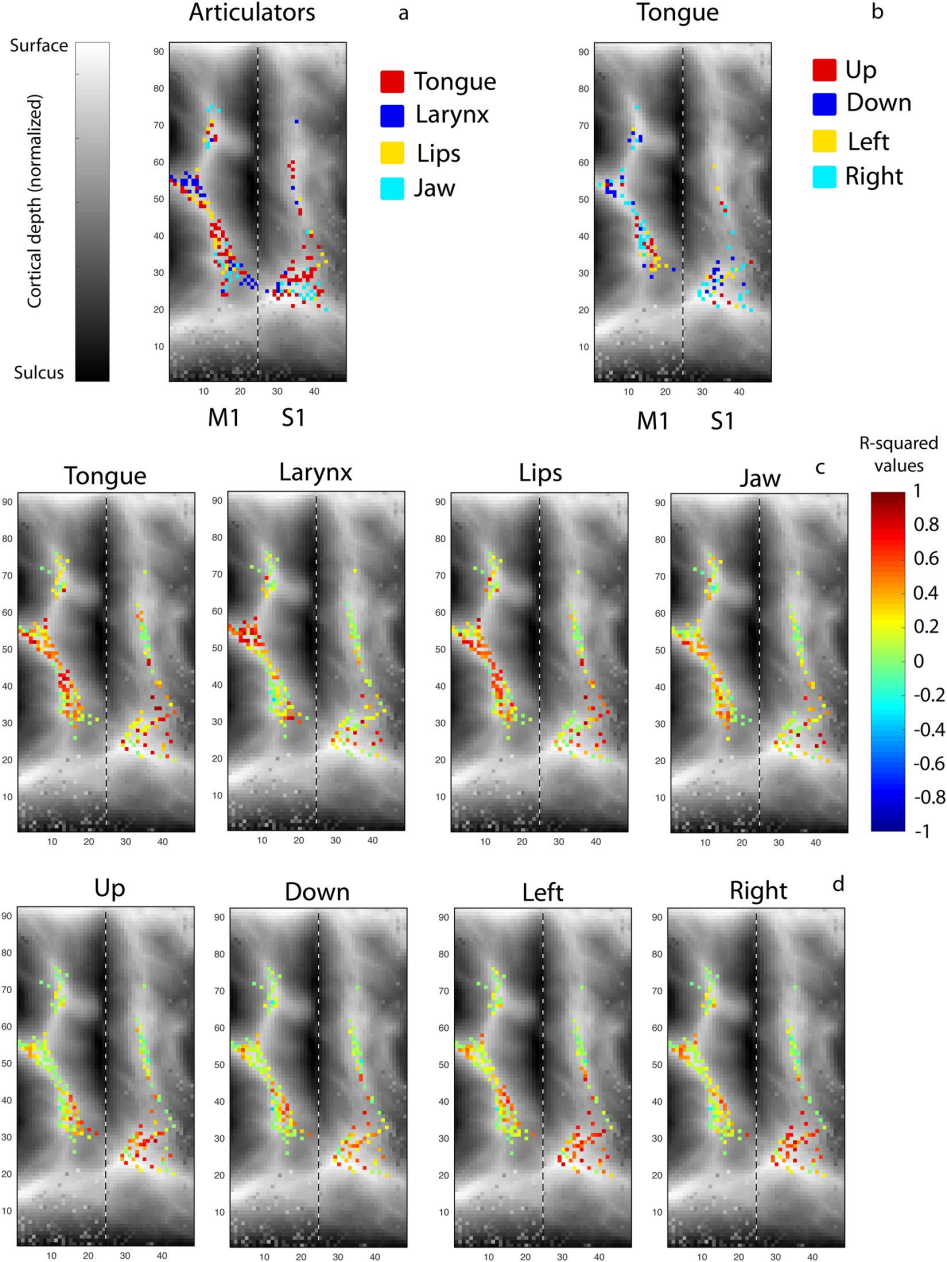
Representations of neural responses during different movements. In the top panels (**a,b**), the average representation of different articulator and tongue movements is shown on an inflated and normalized brain surface with darker colors indicating the sulcus and lighter colors indicating the surface (for anatomical reference of these matrices see Bruurmijn *et al*.^36,37^). Each colored square indicates one electrode. The left side of each plot indicates the precentral gyrus (M1) and the right side the postcentral gyrus (S1), also for subject D in whom the right hemisphere was recorded. The dashed line indicates the central sulcus. Colors indicate for each electrode which movement had the highest r^2^-value in a winner takes all fashion. For the top panel plots (**a,b**), electrodes with an r^2^-value less than 0.2 are not shown as they were not included in the winner-takes-all analysis. The coordinates on the x-axis and y-axis indicate the number of ‘tiles’ in the anterior-posterior and ventral-dorsal direction, respectively. Each tile represents about 1 mm of inflated cortex. In the lower panels (**c,d**), the average of the r^2^-values per movement are shown. Results are based on the first run of each participant and task. See Supplementary Fig. S6 for the data per participant for all runs.

For each articulator movement and tongue movement direction, we investigated where on the sensorimotor cortex the related activity was located (Fig. 5; for individual subject maps see Supplementary Fig. S6). For both the different articulator movements and the different tongue movement directions, we found much overlap. Yet, there seemed to be a topographical organization of articulator representation, with a ventral to dorsal representation of the jaw, larynx, tongue, lips and again larynx. For the different tongue movements, the topographical ordering was less clear (especially in S1) although within M1 the leftward movements seemed to be located somewhat posterior-ventral whereas rightward movements were located more anterior-dorsal. The upward movements seemed to be somewhat more ventral and the downward movements somewhat more dorsal. Note however, that these results are based only on the first run, since only a limited number of participants performed additional runs, and that not all sampled areas were covered by all subjects.

## Discussion

In this study, we showed that with a very straightforward and robust classification procedure, movements of different articulators can be distinguished from each other using HD-ECoG signals. In addition, we found that different movement directions of the tongue can also be distinguished. Both articulator and tongue movements can be classified from a small patch of cortical area (ca. 1 cm^2^). Accurate classification was accomplished using only spatial information (spatial classification). Adding time as a feature of the classification (spatio-temporal classification) did not lead to much higher classification accuracies. This may be explained by a jitter in the onset times of movements within the same class and in the associated neural responses. Correcting for variations in neural activity onset may potentially lead to even better classification than we currently obtained (see for instance^7^). Finally, visual inspection of the activity patterns for each articulator movement and tongue movement direction suggests a topographical ordering, most clearly for the movements of different articulators, with a ventral to dorsal ordering of the jaw, larynx, the tongue, lips and a second area for larynx, respectively.

Overall, the repeated runs yielded similar classification results, although the classification scores of the second run were generally somewhat lower, possibly due to fatigue and associated suboptimal task compliance. Since we were not able to correct for performance (as most movements were invisible from the outside), we cannot rule out that task performance may have caused potential differences in classification accuracy between runs or between subjects. For the articulator movements, subject A indicated to sometimes have difficulty making the movements in time, which may explain partly why the scores were somewhat lower for that subject than for the other subjects. Alternatively, differences in electrode coverage may be associated with variability in classification accuracy between subjects. Indeed, for the two subjects (A & B) who performed one run of tongue movements outside the mouth, there was no noticeable difference in performance and both participants were very accurate, but classification accuracy was substantially lower in subject A. Further investigations as to where these inter-subject differences originate are needed. Since there is a correspondence between ECoG broadband activity and fMRI BOLD responses^38,39^ and since indeed the current results correspond well with earlier fMRI results^18^, pre-surgical fMRI investigations may elucidate what factors influence inter-subject differences in classification results.

Electrodes that were most informative for distinguishing different articulators covered a larger area of the sensorimotor cortex and were less clustered than those most informative for the classification of tongue movements, which were somewhat clustered in the central aspects of the SMC (similar as in^40^). Yet, a large cortical area is not required for high classification scores: accurate classification was obtained from areas of approximately 1 cm^2^. This indicates that small patches of the sensorimotor cortex contain information about movements of different articulators or different movement directions of the tongue. The area that *can* be used for grid placement leading to accurate classification was more concise for tongue movements than for articulator movements. Importantly however, our results suggest that classification of both different articulator movements and different tongue movement directions can be accurate from small cortical areas in similar locations.

The current results are in agreement with a previous fMRI study that showed that lip, tongue, jaw and larynx movements can be distinguished from each other based on sensorimotor cortex activity^18^, albeit with a different recording modality and less cortical coverage (limited to the skull-lining surface). In addition, our finding that different tongue movement directions have distinguishable representations agrees with single cell recordings in primates that have shown different neurons to be related to different movements of the tongue. Stimulation of some neurons for instance resulted in tongue protrusion whereas stimulation of other neurons resulted in retraction^41^. Furthermore, some neurons showed significantly different firing rates for tongue protrusion in different directions^42^. Here we extend those results by showing that different movement directions can be measured from the human SMC using surface electrode recordings of relatively large neuronal ensembles. These results also agree with previous investigations that suggest that, during speech, different positions of the same articulator (i.e. the tongue) lead to different SMC activity patterns^19,20^. For speech-BCI applications it is important to target the cortical areas that contain information about speech movements of both between and within articulators. The current results may benefit the development of speech-BCIs by providing insights in where and how neural activity of different articulators and different movements of the same articulator are represented in the SMC. Furthermore, in agreement to earlier fMRI studies^43^ we found that distinctive information about articulator movements and tongue movement directions can be extracted from both the left and right hemisphere, suggesting that the representation of articulator movements is bilateral.

We found significant overlap in the location of neural activation associated with the movements of different articulators. Visual inspection of the group activity patterns, however, revealed a ventral-dorsal topographical ordering in the neural activity hotspots for the different articulator movements, with the jaw and larynx mostly ventral, the tongue superior to that and the lips and a second area for larynx mostly dorsal. Note however, that these results are based on a group map in which not all areas are equally covered by all subjects and that these results are open for interpretation. These results are similar, however, to those of previous studies with respect to the notion of the two larynx areas^25,44^ and the localization of the tongue in the middle of the vSMC^25,35,44^. Jaw-related activity, however, was found along the central sulcus with the highest activity mostly ventral, while some other studies found this to be more dorsal to the tongue area^25,35^. This discrepancy may be caused by methodological differences: in our study, people had to clench the jaw, while in the other studies, the jaw was opened instead of closed. Stimulation results^44^ indeed support the notion of jaw activity along the central sulcus, also ventrally, similar to what we found. For the lips, we found a similar location with respect to the ventral-dorsal ordering as a previous study^25^, but the activity hotspot was more in the precentral gyrus in our study, whereas Bouchard *et al*.^25^ found it to be more in the postcentral gyrus. Again, task differences may have caused this discrepancy: we used lip protrusion whereas Bouchard *et al*.^25^ used speech movements. In conclusion, the location of neural activity associated with non-speech articulator movements demonstrated in the current study corresponds well to that of speech movements shown in earlier work. Therefore, the current results may be beneficial also for speech-BCI applications that aim to target cortical areas that contain information about different articulators.

For the different tongue movements, the topographical representation was less clear than for the articulators (especially in S1) and we found a large overlap in the neural patterns for different tongue movements. However, although the results may have been driven more by some subjects than by others due to differences in the sampled area (see also Supplementary Fig. S6), there seemed to be some spatial ordering of the representation of different directions of tongue movement within M1. Future research with more participants may indicate if a topographical ordering for different tongue directions indeed exists in the SMC.

The current results are of interest for the development of implantable BCIs in general and speech BCIs in particular. First, a fundamental issue in the development of implantable BCIs is to define the areas that need to be targeted for the system to perform accurately, and without confounds of responding to external stimuli (e.g. auditory). Our results provide insight in where information about different articulators and different tongue movements are most strongly represented and estimate that with small electrode grids in the SMC region (ca. 1 cm^2^), we are able to get high classification results. These results are important since a smaller implant poses significantly less risk and burden of the surgical procedure^45^. Obviously, with a smaller grid, precise placement becomes increasingly important. Preoperative fMRI may be used to define the optimal target location. This method has been successfully used previously for hand movements^10^ and visual responses^46^. Whether or not fMRI is a suitable technique to localize the target locations for a speech BCI remains to be determined. In addition, since the hotspots of articulator movement neural activity can be relatively small (ca. 1 cm), we would like to stress the importance of HD-electrode grids for classification of articulator movements. Clinical electrode grids typically have an interelectrode distance of 1 cm, which leads to significant spatial under-sampling, which most likely hampers our ability to distinguish different articulator movements from one another. Indeed, the importance of HD-grids has also been pointed out in previous speech-BCI studies^14,15^. Second, our results suggest that multiple degrees of freedom can be extracted from a small cortical area, which might potentially benefit current BCI systems. A previously described home based BCI system by Vansteensel and colleagues^10^ for instance, contrasts SMC activity during attempted hand movement against rest and has been shown to be very reliable^10^. However, this system makes use of simple control signals (1 degree of freedom) which limits the speed of communication (2–3 letters per minute). Here we showed that a system such as described by Vansteensel and colleagues^10^ may be extended with more degrees of freedom by using vSMC activity related to articulator and tongue movements. This may potentially increase the control speed of such BCIs. Finally, a recent study reported encouraging decoding of speech from perisylvian areas including premotor, SMC and temporal regions^17^. The current study focused on SMC (to exclude contributions of auditory processing of own and other’s speech) and on discrimination of discrete movements. The complementary findings encourage further investigation into region-specific, high-density ECoG grids combined with advanced decoding approaches^17,47^ for restoring communication in LIS.

It is important to note that replication of these experiments with attempted movements is needed since current results might be influenced by, for instance, sensory feedback which is not present for attempted movements. Earlier studies on this topic with hand movements, however, showed that indeed neural signal changes associated with attempted movements correspond well to those of actual movements, despite the absence of sensory feedback^36,48^. This suggests that the current results bear relevance for BCI applications where no sensory feedback is available. Finally, it is important to test how the current results relate to real time applications in which subjects receive feedback on the classification performance. It has been suggested for instance that neural feedback may improve classification accuracies^49,50^.

## Conclusion

We demonstrate here that with high-density subdural ECoG recordings from the sensorimotor cortex surface, movements of different articulators can be distinguished from each other based on neural activity. In addition, we showed that different movements of one articulator (the tongue) can be classified with high accuracy. Both between and within articulator movements can be classified from a very small cortical area (ca. 1 cm^2^). These findings are of relevance for the development of speech-BCIs that aim to provide people with severe paralysis a muscle-independent tool for communication.

## Supplementary information

SUPPLEMENTARY INFO

## Data Availability

Data can be made available upon request through a data sharing contract.

## References

[1] Levelt, W. J. M. *Speaking: From Intention to Articulation*. (MIT Press, 1993).

[2] Meister IG, Wilson SM, Deblieck C, Wu AD, Iacoboni M (2007). The Essential Role of Premotor Cortex in Speech Perception. Curr. Biol..

[3] Guenther, F. H. & Hickok, G. Chapter 9 - Role of the auditory system in speech production. in *Handbook of* Clinical *Neurology* (eds. Aminoff, M. J., Boller, F. & Swaab, D. F.) **129**, 161–175 (Elsevier, 2015). 10.1016/B978-0-444-62630-1.00009-3 25726268

[4] American Congress of Rehabilitation Medicine (1995). Recommendations for use of uniform nomenclature pertinent to patients with severe alterations in consciousness. Arch. Phys. Med. Rehabil..

[5] Posner, J. B., Plum, F., Saper, C. B. & Schiff, N. *Plum and Posner’s Diagnosis of Stupor and Coma*. (Oxford University Press, USA, 2007).

[6] Smith E, Delargy M (2005). Locked-in syndrome. BMJ.

[7] Branco MP (2017). Decoding hand gestures from primary somatosensory cortex using high-density ECoG. NeuroImage.

[8] Hochberg LR (2012). Reach and grasp by people with tetraplegia using a neurally controlled robotic arm. Nature.

[9] Collinger JL (2013). High-performance neuroprosthetic control by an individual with tetraplegia. The Lancet.

[10] Vansteensel MJ (2016). Fully Implanted Brain–Computer Interface in a Locked-In Patient with ALS. N. Engl. J. Med..

[11] Kellis S (2010). Decoding spoken words using local field potentials recorded from the cortical surface. J. Neural Eng..

[12] Brumberg JS, Wright EJ, Andreasen DS, Guenther FH, Kennedy PR (2011). Classification of Intended Phoneme Production from Chronic Intracortical Microelectrode Recordings in Speech-Motor Cortex. Front. Neurosci..

[13] Mugler EM (2014). Direct classification of all American English phonemes using signals from functional speech motor cortex. J. Neural Eng..

[14] Herff C (2015). Brain-to-text: decoding spoken phrases from phone representations in the brain. Neural Technol..

[15] Ramsey NF (2018). Decoding spoken phonemes from sensorimotor cortex with high-density ECoG grids. NeuroImage.

[16] Pei X, Barbour DL, Leuthardt EC, Schalk G (2011). Decoding vowels and consonants in spoken and imagined words using electrocorticographic signals in humans. J. Neural Eng..

[17] Anumanchipalli GK, Chartier J, Chang EF (2019). Speech synthesis from neural decoding of spoken sentences. Nature.

[18] Bleichner MG (2015). Classification of mouth movements using 7 T fMRI. J. Neural Eng..

[19] Salari E, Freudenburg ZV, Vansteensel MJ, Ramsey NF (2018). The influence of prior pronunciations on sensorimotor cortex activity patterns during vowel production. J. Neural Eng..

[20] Chartier J, Anumanchipalli GK, Johnson K, Chang EF (2018). Encoding of Articulatory Kinematic Trajectories in Human Speech Sensorimotor Cortex. Neuron.

[21] Booij, G. *The Phonology of Dutch*. (Clarendon Press, 1999).

[22] Rietveld, A. & van Heuven, V. *Algemene Fonetiek*. (Coutinho, 2001).

[23] Wang, J., Green, J. R. & Samal, A. Individual articulator’s contribution to phoneme production. In *2013 IEEE International Conference on Acoustics, Speech and Signal Processing (ICASSP)* 7785–7789, 10.1109/ICASSP.2013.6639179 (2013).

[24] Crone NE, Miglioretti DL, Gordon B, Lesser RP (1998). Functional mapping of human sensorimotor cortex with electrocorticographic spectral analysis. II. Event-related synchronization in the gamma band. Brain.

[25] Bouchard KE, Mesgarani N, Johnson K, Chang EF (2013). Functional organization of human sensorimotor cortex for speech articulation. Nature.

[26] Manning JR, Jacobs J, Fried I, Kahana MJ (2009). Broadband shifts in LFP power spectra are correlated with single-neuron spiking in humans. J. Neurosci. Off. J. Soc. Neurosci..

[27] Miller KJ, Sorensen LB, Ojemann JG, Nijs M (2009). den. Power-Law Scaling in the Brain Surface Electric Potential. PLOS Comput. Biol..

[28] Ray S, Maunsell JHR (2011). Different Origins of Gamma Rhythm and High-Gamma Activity in Macaque Visual Cortex. PLOS Biol..

[29] Hermes D, Miller KJ, Noordmans HJ, Vansteensel MJ, Ramsey NF (2010). Automated electrocorticographic electrode localization on individually rendered brain surfaces. J. Neurosci. Methods.

[30] Branco MP (2018). ALICE: A tool for automatic localization of intra-cranial electrodes for clinical and high-density grids. J. Neurosci. Methods.

[31] Branco MP, Leibbrand M, Vansteensel MJ, Freudenburg ZV, Ramsey NF (2018). GridLoc: An automatic and unsupervised localization method for high-density ECoG grids. NeuroImage.

[32] Salari E, Freudenburg ZV, Vansteensel MJ, Ramsey NF (2018). Spatial-Temporal Dynamics of the Sensorimotor Cortex: Sustained and Transient Activity. IEEE Trans. Neural Syst. Rehabil. Eng..

[33] Bruns A (2004). Fourier-, Hilbert- and wavelet-based signal analysis: are they really different approaches?. J. Neurosci. Methods.

[34] Branco MP, Freudenburg ZV, Aarnoutse EJ, Vansteensel MJ, Ramsey NF (2018). Optimization of sampling rate and smoothing improves classification of high frequency power in electrocorticographic brain signals. Biomed. Phys. Eng. Express.

[35] Grabski K (2012). Functional MRI assessment of orofacial articulators: Neural correlates of lip, jaw, larynx, and tongue movements. Hum. Brain Mapp..

[36] Bruurmijn MLCM, Pereboom IPL, Vansteensel MJ, Raemaekers MAH, Ramsey NF (2017). Preservation of hand movement representation in the sensorimotor areas of amputees. Brain.

[37] Bruurmijn, M. L. C. M., Schellekens, W., Raemaekers, M. A. H. & Ramsey, N. F. A Novel 2d Standard Cartesian Representation for the Human Sensorimotor Cortex. *Neuroinformatics* (In press). 10.1007/s12021-019-09441-y PMC7083812 31797264

[38] Hermes D (2012). Neurophysiologic correlates of fMRI in human motor cortex. Hum. Brain Mapp..

[39] Siero JC (2014). BOLD matches neuronal activity at the mm scale: A combined 7 T fMRI and ECoG study in human sensorimotor cortex. NeuroImage.

[40] Schalk G (2008). Real-time detection of event-related brain activity. NeuroImage.

[41] Yao D (2002). Neuronal Activity Patterns in Primate Primary Motor Cortex Related to Trained or Semiautomatic Jaw and Tongue Movements. J. Neurophysiol..

[42] Murray GM, Sessle BJ (1992). Functional properties of single neurons in the face primary motor cortex of the primate. III. Relations with different directions of trained tongue protrusion. J. Neurophysiol..

[43] Bunton K (2008). Speech versus Nonspeech: Different Tasks, Different Neural Organization. Semin. Speech Lang..

[44] Penfield W, Boldrey E (1937). Somatic motor and sensory representation in the cerebral cortex of man as studied by electrical stimulation. Brain J. Neurol..

[45] Wong CH (2009). Risk factors for complications during intracranial electrode recording in presurgical evaluation of drug resistant partial epilepsy. Acta Neurochir. (Wien).

[46] Zhang D (2013). Toward a minimally invasive brain–computer interface using a single subdural channel: A visual speller study. NeuroImage.

[47] Angrick M (2019). Speech synthesis from ECoG using densely connected 3D convolutional neural networks. J. Neural Eng..

[48] Blokland, Y. *et al*. Detection of event-related desynchronization during attempted and imagined movements in tetraplegics for brain switch control. In *2012 Annual International Conference of the IEEE Engineering in Medicine and Biology Society (EMBC)* 3967–3969, 10.1109/EMBC.2012.6346835 (2012). 10.1109/EMBC.2012.6346835 23366796

[49] Wolpaw JR, Birbaumer N, McFarland DJ, Pfurtscheller G, Vaughan TM (2002). Brain–computer interfaces for communication and control. Clin. Neurophysiol..

[50] Taylor DM, Tillery SIH, Schwartz AB (2002). Direct Cortical Control of 3D Neuroprosthetic Devices. Science.

