# Supplementary material for: Classification of Articulator Movements and Movement Direction from Sensorimotor Cortex Activity
Source: Sci Rep. 2019 Oct 2;9:14165. doi: 10.1038/s41598-019-50834-5 (PMC6775133; doi:10.1038/s41598-019-50834-5)
Supplement: Supplementary file 1 — SUPPLEMENTARY INFO [file 41598_2019_50834_MOESM1_ESM.docx]

**Classification of Articulator Movements and Movement Direction from Sensorimotor Cortex Activity**

**Supplementary material**

*E. Salari, Z.V. Freudenburg, M.P. Branco, E.J. Aarnoutse, M.J. Vansteensel, N.F. Ramsey**

*UMC Utrecht Brain Center, Department of Neurology and Neurosurgery, University Medical Center Utrecht, Utrecht, The Netherlands*

*Corresponding author: N.F. Ramsey

Phone: +31 (0)88 755 6862

Fax: +31 (0)30 254 2100

E-mail: n.f.ramsey@umcutrecht.nl

Address: Heidelberglaan 100, 3508 GA, Utrecht, The Netherlands.

Room G03 1.24, P.O. Box 85500

ORCID:

E. Salari: 0000-0002-7467-3297

Z. Freudenburg: 0000-0002-2790-0020

M. Branco: 0000-0002-7316-8846

E. Aarnoutse: 0000-0001-7648-250X

M. Vansteensel: 0000-0002-9252-5116

N. Ramsey: 0000-0002-7136-259X

**
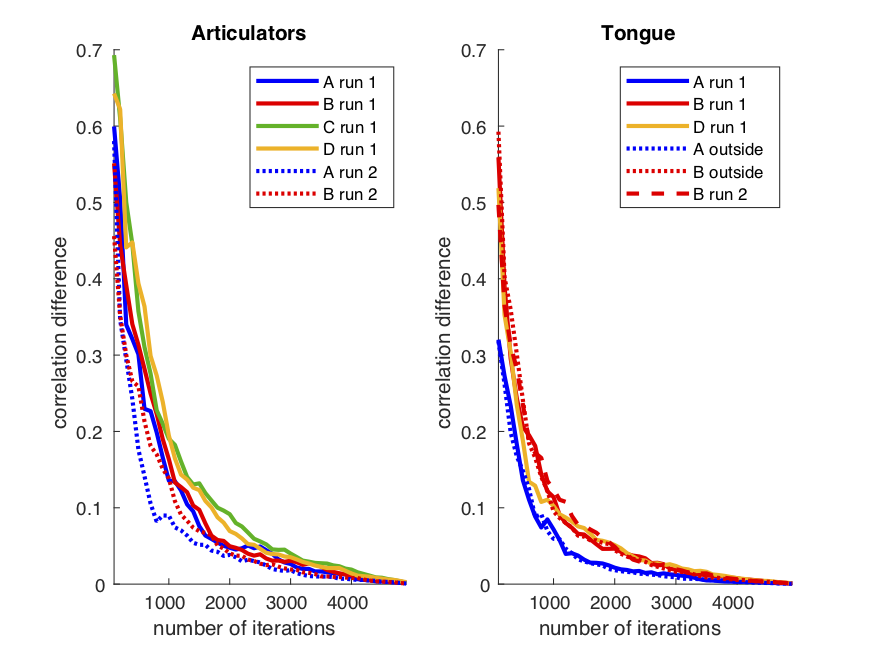
**

**Fig. S1 Random search stabilization.** The stabilization of the random search result in relation to the number of iterations (x-axis) is shown for each participant and run. For each iteration, the pattern of most informative electrodes is calculated. On the y-axis, the average difference in correlation of each pattern with the subsequent patterns is show.


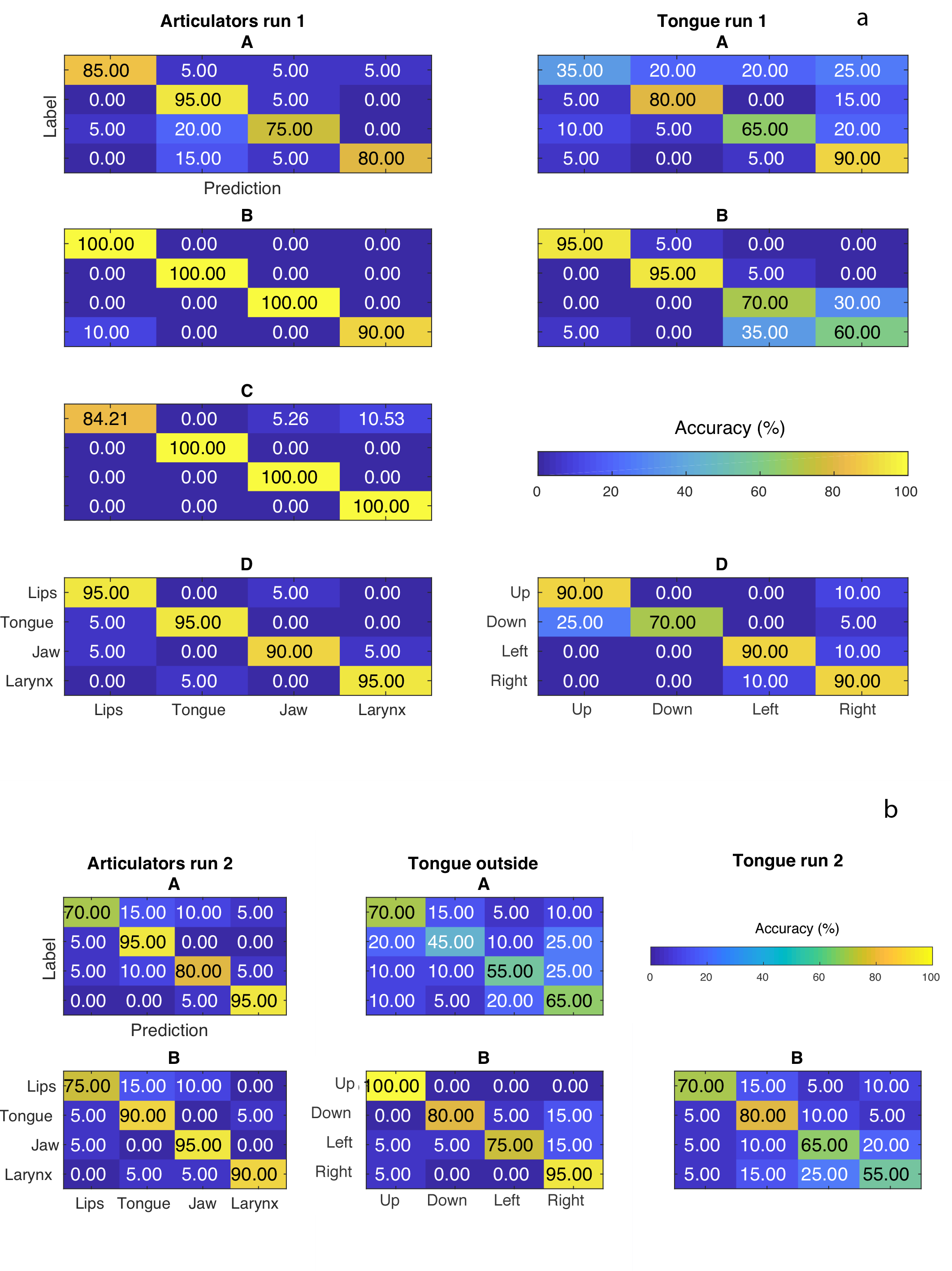


**Fig. S2 Confusion matrices of the spatial classification.** The confusion matrixes are shown for each subject of the first run (a) and for the subjects that performed additional runs (b). Colors indicate the percentage of trials classified within each class.


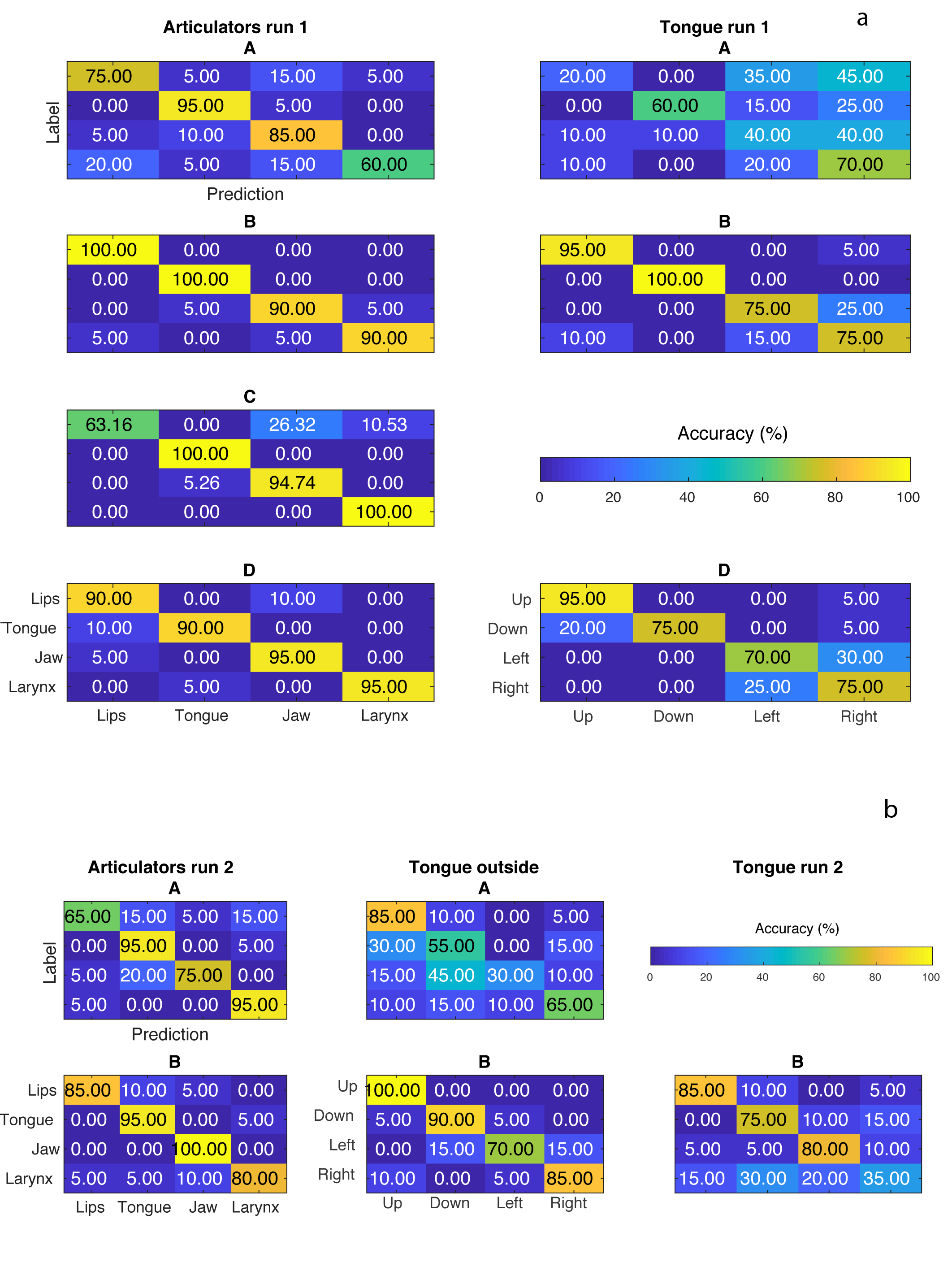


**Fig. S3 Confusion matrices of the spatio-temporal classification.** The confusion matrixes are shown for each subject of the first run (a) and for the subjects that performed additional runs (b). Colors indicate the percentage of trials classified within each class.


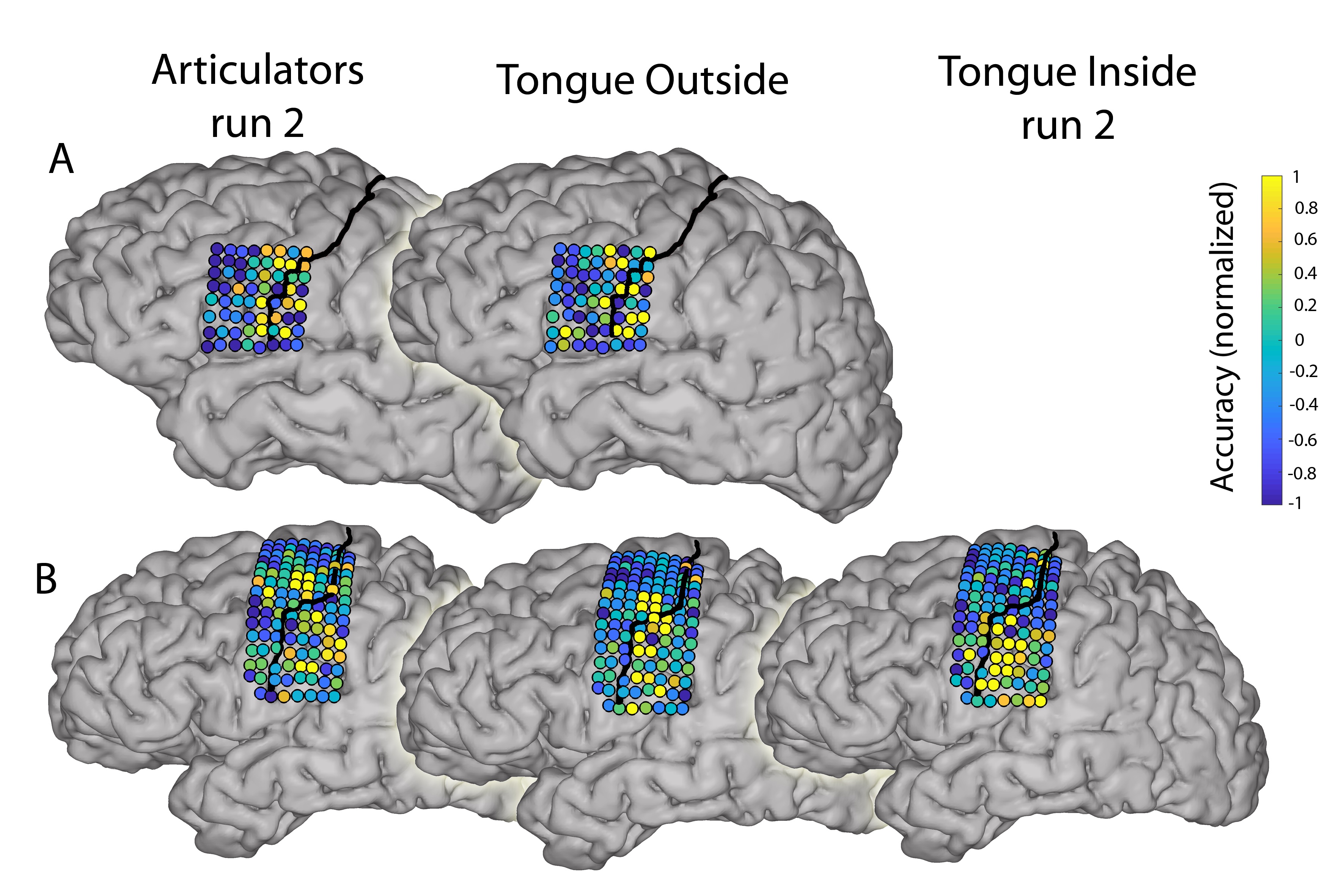


**Fig. S4 Most informative electrodes for additional runs.** The most informative electrodes are shown in color for each subject (A & B) and for each task. Informative electrodes were determined by a random search procedure. Colors indicate the normalized average accuracy score of this procedure. The warmer the color the higher the classification was on average if that electrode was included.


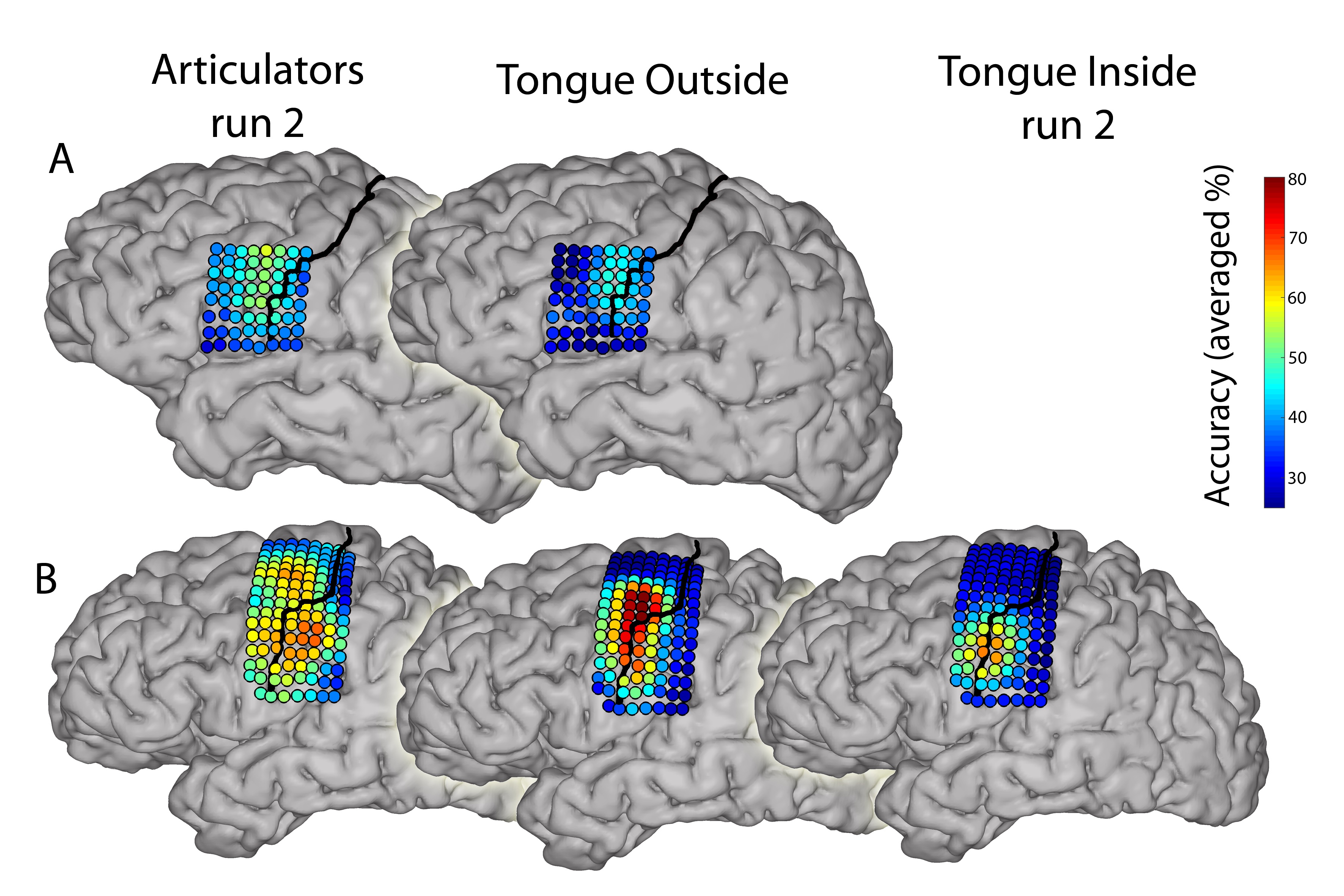


**Fig. S5 Search light results for additional runs.** The most informative areas are shown in color for each subject (A & B) and for each task. Informative areas were determined by a search light procedure (a 3x3 grid size was used for the current figure). For this plot, we choose a search light of three electrode rows and columns, corresponding to approximately 12x12 mm for subject A & B. Colors indicate the normalized average accuracy score of this procedure.

**
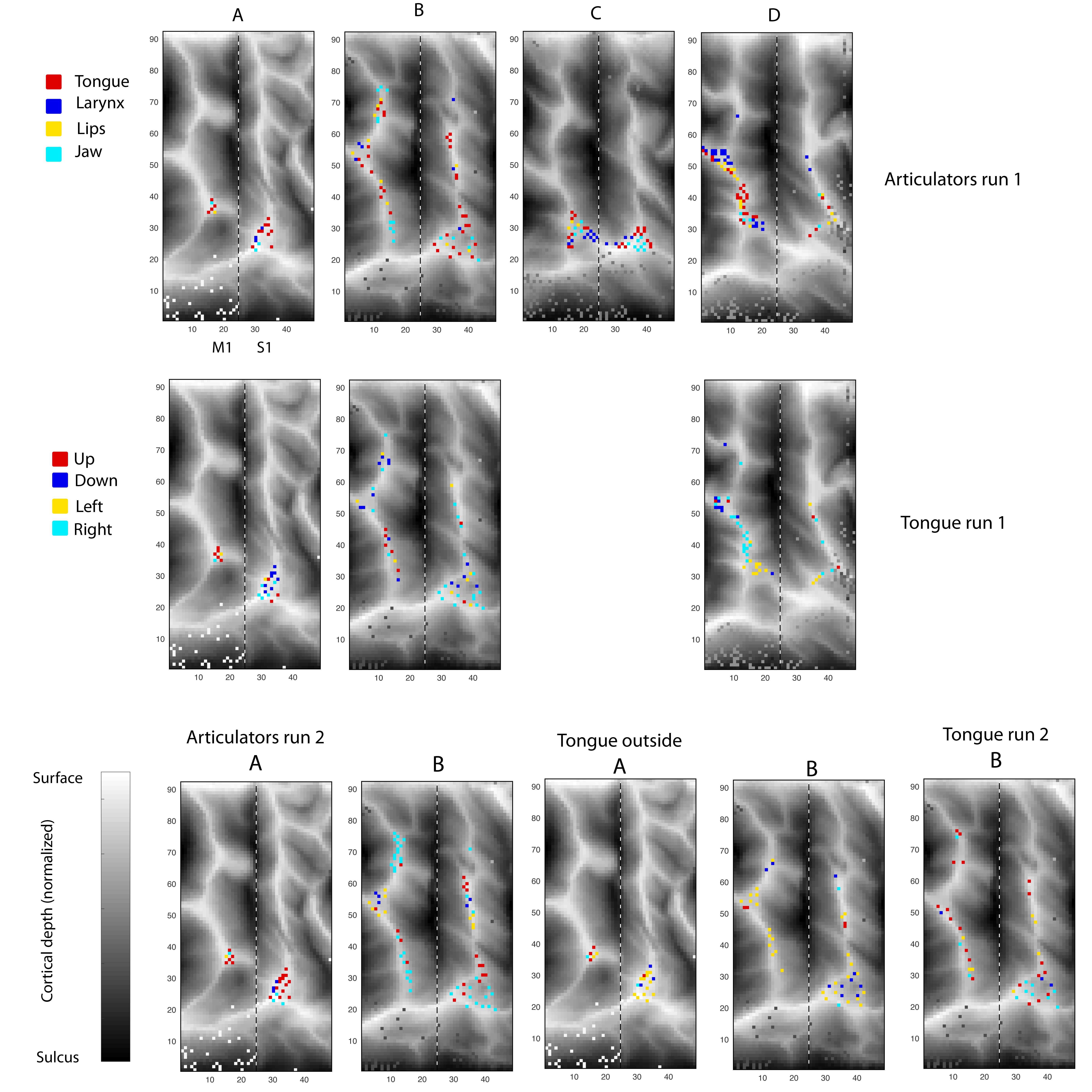
**

**Fig. S6 Localization of different movements per subject and per run.** Each colored square indicates one electrode on an inflated and normalized brain surface. Darker colors indicating the sulcus and lighter colors indicating the surface (for anatomical reference of these matrices see ^1,2^. The left side of each plot indicates the precentral gyrus (M1) and the right side the postcentral gyrus (S1). The dashed line indicates the central sulcus. Colors indicate for each electrode which movement had the highest r^2^-value in a winner takes all fashion. Electrodes with an r^2^-value less than 0.2 are not shown. The coordinates on the x-axis and y-axis indicate the number of ‘tiles’ in the anterior-posterior and ventral-dorsal direction, respectively. Each tile represents about 1 mm of inflated cortex.

**References**

1. Bruurmijn, M. L. C. M., Schellekens, W., Raemaekers, M. A. H. & Ramsey, N. F. A NOVEL 2D STANDARD CARTESIAN REPRESENTATION FOR THE HUMAN SENSORIMOTOR CORTEX. *Neuroinformatics* (in press).

2. Bruurmijn, M. L. C. M., Pereboom, I. P. L., Vansteensel, M. J., Raemaekers, M. A. H. & Ramsey, N. F. Preservation of hand movement representation in the sensorimotor areas of amputees. *Brain* **140**, 3166–3178 (2017).
